# Bringing credibility to observational research in critical care: the case of target trial emulation designs

**DOI:** 10.62675/2965-2774.20250142

**Published:** 2025-08-08

**Authors:** Sérgio Renato da Rosa Decker, Ary Serpa

**Affiliations:** 1 Hospital Moinhos de Vento Department of Internal Medicine Porto Alegre RS Brazil Department of Internal Medicine, Hospital Moinhos de Vento - Porto Alegre (RS), Brazil.; 2 Smith Center for Outcomes Research Beth Israel Deaconess Medical Center and Harvard Medical School Boston United States Smith Center for Outcomes Research, Beth Israel Deaconess Medical Center and Harvard Medical School- Boston, United States.; 3 Monash University Australian and New Zealand Intensive Care Research Centre School of Public Health and Preventive Medicine Melbourne Australia Australian and New Zealand Intensive Care Research Centre, School of Public Health and Preventive Medicine, Monash University- Melbourne, Australia.; 4 Austin Hospital Department of Intensive Care Melbourne Australia Department of Intensive Care, Austin Hospital - Melbourne, Australia.; 5 University of Melbourne Melbourne Medical School Department of Critical Care Melbourne Australia Department of Critical Care, Melbourne Medical School, University of Melbourne, Austin Hospital - Melbourne, Australia.; 6 Hospital Israelita Albert Einstein Department of Critical Care Medicine São Paulo SP Brazil Department of Critical Care Medicine, Hospital Israelita Albert Einstein - São Paulo (SP), Brazil.

## INTRODUCTION

Historically, relying on observational data to evaluate causal effects has been considered a sin, with journals often advising authors to remove causal language from their results, whereas randomized clinical trials remain the gold standard for assessing the comparative effectiveness and safety of treatments.^( 1 )^ However, clinical trials are often impractical, unethical, or untimely.^( 2 )^ In addition, in recent years, clinical trials in the critical care field have frequently yielded negative results or reported findings that were not confirmed in subsequent studies.^( 3 )^ One of the leading hypotheses is that the eligible and included population in critical care trials is not representative of the population that might benefit most from the therapy, or does not reflect the target population that is most likely to receive the therapy in real-world clinical practice.^( 3 )^

The empirical application of various statistical methods developed in epidemiology and econometrics over the past century, combined with the increasingly common availability of large databases (such as electronic health records, surveys, administrative data, and data from mobile or wearable devices), has reshaped the role of observational studies in drawing causal conclusions.^( 4 , 5 )^ More recently, Hernán and Robins pioneered the development of a causal framework that structures observational data analysis into a well-defined protocol, mimicking the design of a randomized clinical trial: the target trial emulation framework (i.e., the emulation of a hypothetical randomized clinical trial designed to answer a specific causal question).^( 4 - 6 )^ This approach has gained popularity, leading high-impact journals to reconsider their stance on reporting causal conclusions derived from observational data.^( 7 , 8 )^

In a recent publication of Critical Care Science, the study by Serpa Neto et al. exemplified the strengths of the target trial emulation framework for comparative effectiveness using observational data in critical care research.^( 9 )^ Particularly, the authors explicitly specified the hypothetical randomized trial that would answer the causal question of interest and how they emulated the trial using observational data (Table 1S - Supplementary Material). Additionally, the authors carefully synchronized the timing of eligibility assessment, treatment assignment, and start of follow-up to minimize bias. Finally, the use of a g-formula approach effectively adjusted for time-dependent confounding and increased the validity of causal inference by approximating the results to the "per-protocol" effect of dexmedetomidine under ideal adherence conditions.

On the basis of this new and potentially transformative design for critical care research, which increases the credibility of observational studies when comparing treatment strategies, we highlight three points that readers from a clinical audience should carefully consider when assessing the reliability of target trial emulation (or observational study) results.

## SHOULD I TRUST THE RESULTS OF CAUSAL EFFECTS FROM OBSERVATIONAL STUDIES?

First, it is essential to assess whether the authors clearly state the causal question of interest and if they specify the target trial being emulated with observational data to answer that question. As Hernán et al. stated, "the most impactful contribution of the target trial framework is the reduction in the ambiguity of causal questions because we cannot provide valid answers if we do not know what the question is".^( 2 )^ Clearly defining the target trial that would answer the causal question and how to emulate the trial with observational data also helps identify potential sources of bias that may influence the results.^( 2 )^

**Figure 1:**
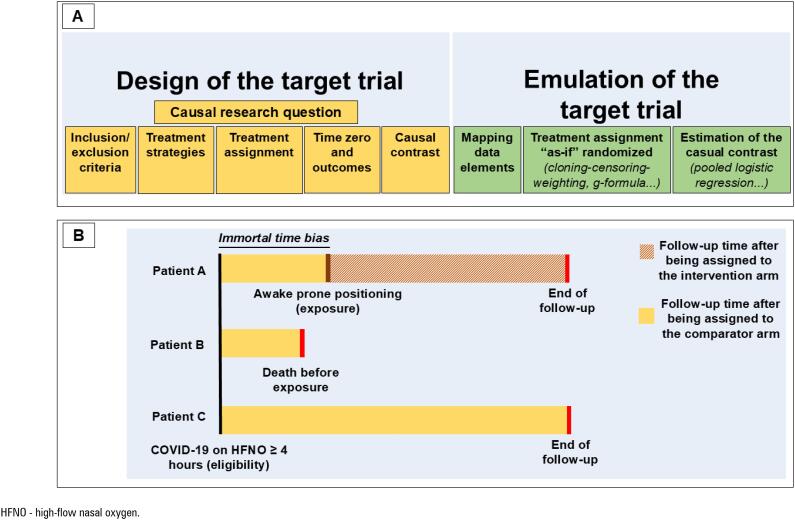
Target trial emulation framework and selection bias in comparative effectiveness studies using observational data.

Two well-known cases illustrate the importance of this approach, as they led to major changes in findings simply by explicitly stating the causal question and specifying the target trial. The apparent beneficial effects of hormone therapy on coronary heart disease and statins on cancer, initially suggested by observational studies, have not been confirmed in randomized trials.^( 6 , 10 )^ While this discrepancy could be due to limitations in the observational data, the precise articulation of the causal question and how to emulate the target trial using observational data ultimately yielded results consistent with those of randomized trials.^6 , 10^ The essential elements to specify in a target trial emulation include the following ( Figure 1A ): the eligibility criteria, treatment strategies, treatment assignment, outcomes, the start and end of follow-up, and causal contrasts (i.e., intention-to-treat or per-protocol effects).^( 2 )^

In Panel A, the importance of stating the causal question to be answered and the main elements to be specified when describing the target trial to answer the question are shown on the left. In Panel A, the analytical steps when observational data are used to emulate the target trial are shown on the right. Panel B illustrates a common pitfall in observational studies: misaligned time zero and immortal time bias. All patients met the eligibility criteria at the time of receiving high-flow nasal oxygen for ≥ 4 hours. However, follow-up for the intervention group (awake prone positioning) began only after ≥ six hours/day were achieved in the prone position (Patient A), whereas follow-up for the control group began at eligibility (Patients B and C). Consequently, patients who died before reaching the exposure threshold (Patient B) were classified as controls, while those who survived long enough to receive the intervention were included in the exposure group (Patient A). This design introduces immortal time bias (i.e., a period during which the outcome [e.g., death] could not occur in the exposure group by design), leading to a potentially exaggerated treatment effect.

Second, we argue that it is essential for authors to clearly describe how they synchronize eligibility, treatment assignment, and the start of follow-up (i.e., time zero) when using observational data to evaluate causal effects.^( 5 , 11 )^ This ensures that common biases in observational studies, such as immortal time bias or other selection biases, do not distort the results.^( 5 , 11 )^ For example, there is an urgent need for interventions to improve post–intensive care outcomes.^( 12 )^ Well-designed observational studies assessing the effects of interventions that are unlikely to be tested in trials (e.g., rehabilitation programs) may inform clinical practice. However, observational studies in this field have reported results that are likely biased because of selection biases.^( 5 , 6 , 11 , 13 )^ Similarly, in a recent multicenter observational study evaluating the effect of awake prone positioning (AW-PP) in critically ill patients with COVID-19-related acute respiratory failure,^( 14 )^ patients were classified into the AW-PP group if they remained prone for at least six hours/day, with follow-up starting at that point of exposure. In contrast, patients in the control group included those who did not meet this threshold, with follow-up beginning earlier, immediately after the initiation of high-flow nasal oxygen therapy ( Figure 1B ). As a result, patients who were too unstable or who deteriorated before achieving six hours of AW-PP were included in the control group (Patient B, Figure 1B ), whereas those who tolerated AW-PP longer were included in the exposure group (Patient A, Figure 1B ). This inadvertently introduced immortal time bias and created differences in time zero and eligibility criteria between the groups. Consequently, the study may have answered a slightly different question than intended: What is the effect of AW-PP in patients who survive and tolerate at least six hours of prone positioning, compared with that in those who do not? The resulting odds ratio of 0.19 (95%CI 0.10 - 0.28) for intubation is substantially lower and inconsistent with estimates reported in a meta-trial of six randomized clinical trials evaluating the same intervention (hazard ratio, 0.75 [95%CI 0.62 - 0.91]).^( 15 )^ The target trial emulation framework helps avoid such pitfalls by aligning the eligibility criteria, treatment assignment, and start of follow-up across groups.^( 11 )^

The target trial emulation framework addresses this issue by explicitly defining the causal question and the hypothetical trial to be emulated, as outlined above. Additionally, this framework advocates methods such as cloning-censoring-weighting to account for time-related biases.^( 5 , 6 , 11 )^ However, it is important to recognize that in other areas of critical care science, these concerns may be less relevant by design. For example, when strategies for rapid sequence intubation in patients with acute respiratory failure are compared, there is virtually no time between becoming eligible, receiving the treatment strategy, and starting follow-up, which reduces the risk of such biases.

The third and final point concerns the quality of the observational data used to emulate a target trial. Unlike in a randomized trial, where treatment assignment is truly random, in a trial emulation, the assumption of "as-if" randomization is valid only if the database captures all key prognostic factors that could act as confounders for the outcomes of interest.^( 2 )^ For example, the study accompanying this viewpoint has the merit of leveraging natural language processing models to define cohort characteristics, track time-varying covariates, and ascertain exposures and outcomes.^( 9 )^ In this study, the variables evaluated appear sufficient to adjust for confounding when analyzing the primary outcome (assessing whether dexmedetomidine leads to faster resolution of agitation in ICU patients). However, when all-cause mortality was evaluated, despite dexmedetomidine-treated patients being sicker on the basis of measured variables, they had lower crude mortality. This suggests the presence of unmeasured confounders influencing the risk of death. The authors commendably acknowledged this limitation and refrained from drawing causal conclusions about mortality, demonstrating a responsible approach to interpreting observational findings in the presence of potential residual confounding.

## CONCLUSION

By evaluating (i) the clarity of the causal question and the specification of the target trial being emulated with observational data, (ii) the proper alignment of the time points for eligibility ascertainment, treatment assignment, and the start of follow-up, and (iii) the quality of the data and the extent to which available variables account for confounding factors, clinicians can make well-informed judgments on the credibility of target trial emulation results. Additionally, this viewpoint may help critical care researchers improve the reporting and discussion of their observational study results aiming to assess comparative effectiveness, effectively communicating their strengths and limitations to a clinical audience in the years to come.
