# Supplementary material for: Bringing credibility to observational research in critical care: the case of target trial emulation designs
Source: Crit Care Sci. 2025 Aug 8;37:e20250142. doi: 10.62675/2965-2774.20250142 (PMC12614949; doi:10.62675/2965-2774.20250142)
Supplement: Supplementary file 1 [file 2965-2774-ccsci-37-e20250142-suppl01.pdf]

Critical Care Science
https://doi.org/10.62675/2965-2774.20250142

SUPPLEMENTARY MATERIAL

Bringing credibility to observational research in critical care: the case of target trial emulation designs

Sérgio Renato da Rosa Decker[1,2], Ary Serpa Neto[3,4,5,6]

Table 1S - Target trial protocol specification and emulation using observational data. Example of dexmedetomidine to treat agitation in the intensive care unit

Protocol component	Description	Specification	Emulation
**Eligibility criteria**	Who will be included in the study?	Patients who developed agitation during the ICU stay. Patients who started on dexmedetomidine before the episode of agitation, and patients with missing data for in-hospital mortality were excluded	Same as the hypothetical trial
**Treatment strategies**	What interventions will eligible persons receive?	Treatment with dexmedetomidine vs. usual care without the use of dexmedetomidine	Same as the hypothetical trial
**Treatment assignment**	How will eligible persons be assigned to the intervention?	Patients are randomly assigned to one of the strategies at time zero	Assignment as if random given the covariates measured
**Outcomes**	What outcomes will be measured and compared between intervention arms?	30-day resolution of agitation	Same as the hypothetical trial
**Start/ end of follow-up**	What is time 0, and for how much time will eligible persons be followed?	Time 0 is the time at which the patient is assigned to a strategy (and all eligibility criteria are met) and ends at the resolution of agitation, occurrence of death, or 30 days, whichever comes first	Follow-up starts the first time all eligibility criteria are met
**Causal contrast**	Which counterfactual contrasts will be estimated?	Per protocol effect	Observational analog of the per protocol effect, assuming conditional exchangeability*
**Statistical analysis**	What are the methods employed to calculate the causal contrast?	In the per-protocol analysis, patients were censored when they deviated from their assigned strategy	The per-protocol effect was estimated using the parametric g-formula, evaluating the full adherence to the protocol. Different moments of dexmedetomidine initiation after agitation onset were investigated in a sensitivity analysis.

ICU - intensive care unit. * Assumes that baseline and time-varying confounding is known, measured, and accounted for, which commonly do not hold in per-protocol effects in randomized clinical trials.

This is an open access article under the CC BY license (https://creativecommons.org/licenses/by/4.0/).
